# Non-target Effects of Green Fluorescent Protein (GFP)-derived Double-Stranded RNA (dsRNA-GFP) Used in Honey Bee RNA Interference (RNAi) Assays 

**DOI:** 10.3390/insects4010090

**Published:** 2013-01-04

**Authors:** Francis M. F. Nunes, Aline C. Aleixo, Angel R. Barchuk, Ana D. Bomtorin, Christina M. Grozinger, Zilá L. P. Simões

**Affiliations:** 1 Departamento de Genética, Faculdade de Medicina de Ribeirão Preto, Universidade de São Paulo, Ribeirão Preto, São Paulo, 14049-900, Brazil; E-Mail: anadurvalina@usp.br; 2 Departamento de Biologia Celular, Tecidual e do Desenvolvimento, Instituto de Ciências Biomédicas, Universidade Federal de Alfenas, Alfenas, Minas Gerais, 37130-000, Brazil; E-Mail: barchuk@unifal-mg.edu.br; 3 Department of Entomology, Center for Pollinator Research, Huck Institutes of the Life Sciences, Pennsylvania State University, University Park, 16802, Pennsylvania, USA; E-Mail: cmgrozinger@psu.edu; 4 Departamento de Biologia, Faculdade de Filosofia, Ciências e Letras de Ribeirão Preto, Universidade de São Paulo, Ribeirão Preto, São Paulo, 14040-901, Brazil; E-Mail: zlpsimoe@usp.br

**Keywords:** RNAi, dsRNA, GFP, honeybee, *Apis mellifera*, off-target effects

## Abstract

RNA interference has been frequently applied to modulate gene function in organisms where the production and maintenance of mutants is challenging, as in our model of study, the honey bee, *Apis mellifera*. A green fluorescent protein (GFP)-derived double-stranded RNA (dsRNA-GFP) is currently commonly used as control in honey bee RNAi experiments, since its gene does not exist in the *A. mellifera* genome. Although dsRNA-GFP is not expected to trigger RNAi responses in treated bees, undesirable effects on gene expression, pigmentation or developmental timing are often observed. Here, we performed three independent experiments using microarrays to examine the effect of dsRNA-GFP treatment (introduced by feeding) on global gene expression patterns in developing worker bees. Our data revealed that the expression of nearly 1,400 genes was altered in response to dsRNA-GFP, representing around 10% of known honey bee genes. Expression changes appear to be the result of both direct off-target effects and indirect downstream secondary effects; indeed, there were several instances of sequence similarity between putative siRNAs generated from the dsRNA-GFP construct and genes whose expression levels were altered. In general, the affected genes are involved in important developmental and metabolic processes associated with RNA processing and transport, hormone metabolism, immunity, response to external stimulus and to stress. These results suggest that multiple dsRNA controls should be employed in RNAi studies in honey bees. Furthermore, any RNAi studies involving these genes affected by dsRNA-GFP in our studies should use a different dsRNA control.

## 1. Introduction

RNA interference (RNAi) technologies (RNAi) are important tools for manipulating transcript levels and exploring gene function in a wide range of species. RNAi is a particularly critical tool for functional genomic studies in species where other genetic manipulations, such as the development of transgenics or mutant strains, are not feasible [1]. Honey bees (*Apis mellifera*), for example, are an excellent model system for sophisticated studies of cognition, neurobiology, plasticity and complex social behavior, but thus far, the development and maintenance of knockout and transgenic lineages in this species have not been possible [2]. Over the last decade, RNAi has been successfully adopted as the major genetic tool for gene function analysis in honey bees [3,4,5]. However, there can be considerable variability in the levels and duration of transcript knockdowns between tissues, individuals and experiments. In order to take full advantage of this powerful technology to study gene function in honey bees and other insects, it is necessary to develop a complete understanding of the mechanisms by which RNAi decreases transcript abundance and, in particular, to examine and characterize any off-target effects. 

Off-target effects are non-specific and caused by undesired base-pairing of non-target genes with small interfering RNA (siRNA) derived from double-stranded RNA (dsRNA) [6]. Off-target effects can be widespread and can alter expression of large numbers of genes, as previously reported in RNAi experiments involving plants, invertebrates, vertebrates [7,8,9], as well as honey bees [10].

A green fluorescent protein (GFP)-derived dsRNA (dsRNA-GFP) has been used as an exogenous control for RNAi assays in several arthropod species, including *Marsupenaeus japonicus* [11], *Pacifastacus leniusculus* [12], *Spodoptera exigua* [13,14], *Acyrthosiphon pisum* [15], *Aedes aegypti* [16], *Antheraea sp.* [17], *Locusta migratoria* [18], *Schistocerca gregaria* [19], *Bactericerca cockerelli* [20] and *Apis mellifera* [5,21,22,23,24,25,26,27]. Its gene sequence is not found in the honey bee genome. Although dsRNA-GFP is not expected to trigger an RNAi response in treated bees, undesirable effects on gene expression, pupal pigmentation or developmental timing have been routinely observed. To better understand the molecular and phenotypic effects of dsRNA-GFP in honey bees and to evaluate its use as a control for RNAi studies, we examined the impact of dsRNA-GFP on global gene expression patterns in developing workers. The dsRNA-GFP was introduced using a non-invasive feeding protocol [23]. We found that dsRNA-GFP causes large-scale changes in gene expression associated with multiple biological processes. Furthermore, dsRNA-GFP exposure tended to preferentially decrease, rather than increase, expression of genes compared to controls.

## 2. Results and Discussion

The double-stranded RNA for green fluorescent protein (dsRNA-GFP) is widely used as an exogenous control in honey bee RNAi studies [5,21,22,23,24,25,26,27]. In our studies, changes in gene expression, pupal pigmentation or developmental timing are the most frequent undesired effects in RNAi screens that use dsRNA-GFP as control. These effects have been also noted by other bee researchers during discussions related to RNAi approaches at the IUSSI Congress 2010 (Copenhagen, Denmark) and Workshop on Honey Bee Genomics & Biology 2011 (Cold Spring Harbor, USA). Therefore, those observable side effects are recurring and not yet reported in the literature. Based on this, we decided to perform a large-scale gene expression analysis to compare untreated and dsRNA-GFP treated developing workers.

Three technically distinct experiments were conducted in two different laboratories. Similar numbers of up- and downregulated genes were found for experiments 1 and 2. In addition, results from both experiments showed a greater number of down- than upregulated genes (Table 1). On the other hand, expression of only a few genes was altered by dsRNA-GFP in experiment 3, which examined adult workers.

**Table 1:** Number of honey bee genes displaying different expression levels in response to green fluorescent protein-derived double-stranded RNA (dsRNA-GFP) treatment during worker development. Genes with significant expression levels at FDR < 0.5 (Experiment 1) and adjusted p < 0.05 (Experiments 2 and 3) are reported.

Experiment	Upregulated	Downregulated	Total
1	203	591	794
2	239	423	662
3	4	1	5
Total	446	1,015	1,461

Analyses of the downregulated gene set revealed that only five genes appear in both lists of differentially expressed genes obtained in experiments 1 and 2 (Table S1). Based on Gene Ontology terms, they are classified as enzymes or proteins with binding functions involved in cellular metabolic process, such as RNA processing and transport. In RNAi experiments, long dsRNA molecules are cleaved into small interfering RNA duplexes (siRNA) of ~21 nucleotides by Dicer enzyme [28]. If these siRNAs have sequence similarity to additional, non-target transcripts, they can cause degradation of these transcripts [6]. To determine if the decreased transcript abundance of these five genes is due to off-target effects, we aligned the sequences of each of these transcripts with the dsRNA-GFP sequence. For each gene, alignments revealed multiple perfect base-pairing matches ranging from eight to 11 nucleotides-long, as well as regions of imperfect complementarity (File S2). Previous studies have demonstrated that off-target gene silencing can be mediated by 7 nt perfect matches between a siRNA and targets [29] and even by microRNA-like mechanisms [30,31] targeting both coding and untranslated regions [32,33,34]. Moreover, the occurrence of multiple off-target sites is suggested to enhance the disturbances [29]. It is also likely that siRNA molecules generated from dsRNA-GFP saturate cellular RNAi machinery, so that siRNAs compete with endogenous microRNAs [34]. Thus, the expected microRNA action is perturbed, and their natural targets may be upregulated [35].

Nine genes were found to be upregulated in both experiments 1 and 2 (Table S1). This is not unexpected, since siRNA off-target effects were previously described to cause both down- and upregulation in mammals [36]. In our studies, these upregulated genes are involved in immune responses, oxidoreductase activity, aging, cell homeostasis, morphogenesis, response to external stimulus and response to stress. Among them, GB10133 (superoxide dismutase 1) and GB15855 (thioredoxin 2) are members of an antioxidant system [37] potentially involved in detoxication by modifying the chemical structure of xenobiotics, such as dsRNA-GFP. Also, GB10398 codes for a ninjurin-1-like protein, member of the metazoan conserved Ninjurin family. Ninjurins are cell adhesion molecules, and it is known that their mRNA levels increase after injury, infection or stress [38,39]. Finally, two members of the apidaecin gene family (GB13473 and GB17782) related to humoral immunity [40] were also upregulated. Thus, of these nine genes, five appear to be involved in response to infection or stress. RNAi machinery plays an important role in the insect immune system [41] by triggering antiviral responses in response to exogenous dsRNA viruses [42]. Thus, it is plausible that dsRNA-GFP molecules are recognized as a viral infection, culminating in the activation of immune genes, RNAi systems, siRNA production and consequent off-target effects.

No overlapping genes were found between experiments 1 and 3. Four genes are upregulated in both experiments 2 and 3 (Table S1), including GB10708, a gene encoding for immune responsive protein of 30 kDa (IRP30). Honey bee IRP30 gene was previously reported as a non-canonical immune factor found in social hymenopterans upon bacterial challenge or viral infection [43,44,45].

Little dsRNA-GFP effects were observed in the adult bees. It is important to remember that the dsRNA-GFP was offered only once per experiment in the diet of second instar larvae. Regarding experiments with adult workers, sampling occurred ~ 23 days after treatment. Previous works have shown that dsRNA molecules remain intact for several days in *A. mellifera* and produce long lasting effects [4,23]. Therefore, it is unlikely that such molecules have been degraded or eliminated in adults. We believe that over ~ 23 days, the honey bee immune system has become adapted to the presence of this molecule, no longer recognizing it as viral particles.

These results suggest that: (i) RNAi treatment effect is different during worker development, as in different life stages of other insects [46]; (ii) few target genes are primarily affected by dsRNA-GFP off-target effects; (iii) undesired consequences on gene expression and phenotypes observed in bees treated with dsRNA-GFP could be triggered by disturbances in those targets, affecting downstream gene networks in association with particularities of physiological context of each developmental stage. Furthermore, the physiological and developmental context can influence the effects of dsRNA-GFP treatment: in some genes, dsRNA-GFP treatment has opposite effects in the two experiments, *i.e.*, increased expression in experiment 1, decreased expression in experiment 2 or *vice-versa* (Table S2).

Little overlap in the sets of affected genes between the three experiments/developmental timepoints is not entirely surprising. Side effects triggered by dsRNA-GFP seem to be multifactorial. From a single dsRNA, the resulting off-target effects are specific for different cell types or developmental stage, because the sets of expressed (and consequently misregulated) genes are different in each biological context [47]. Even if dsRNA may be recognized as a viral challenge and upregulate non-self RNA sensors and expression of the immune genes [48], we need to take into account that the range of defense responses can be either evolutionary divergent across species [49,50], as well as developmentally modulated [43,51,52,53]. 

Data from all experiments were combined to produce a comprehensive list of potential gene targets whose expression is altered in treatments with dsRNA-GFP (Table S3). In total, 1,461 genes showed significant changes in expression in response to dsRNA-GFP treatment. This raises concerns about using dsRNA-GFP as a control for RNAi experiments, since it suggest that this treatment can cause changes in about 10% of *A. mellifera* genes, undoubtedly triggering associated development, physiological and behavioral changes. Also, it is important to note that there may be possible additional effects on the transcriptome that could not be examined in the microarray, since it undoubtedly does not cover all possible transcripts. Indeed, several novel coding and non-coding genes were only recently discovered and, thus, are not represented in the microarrays.

To understand how our data are useful and applicable to other honey bee studies, we performed a multiple alignment of GFP partial sequences used by other authors for dsRNA-GFP synthesis (File S1), which showed that sequences are very close to each other, suggesting similar responses.

**Figure 1:**
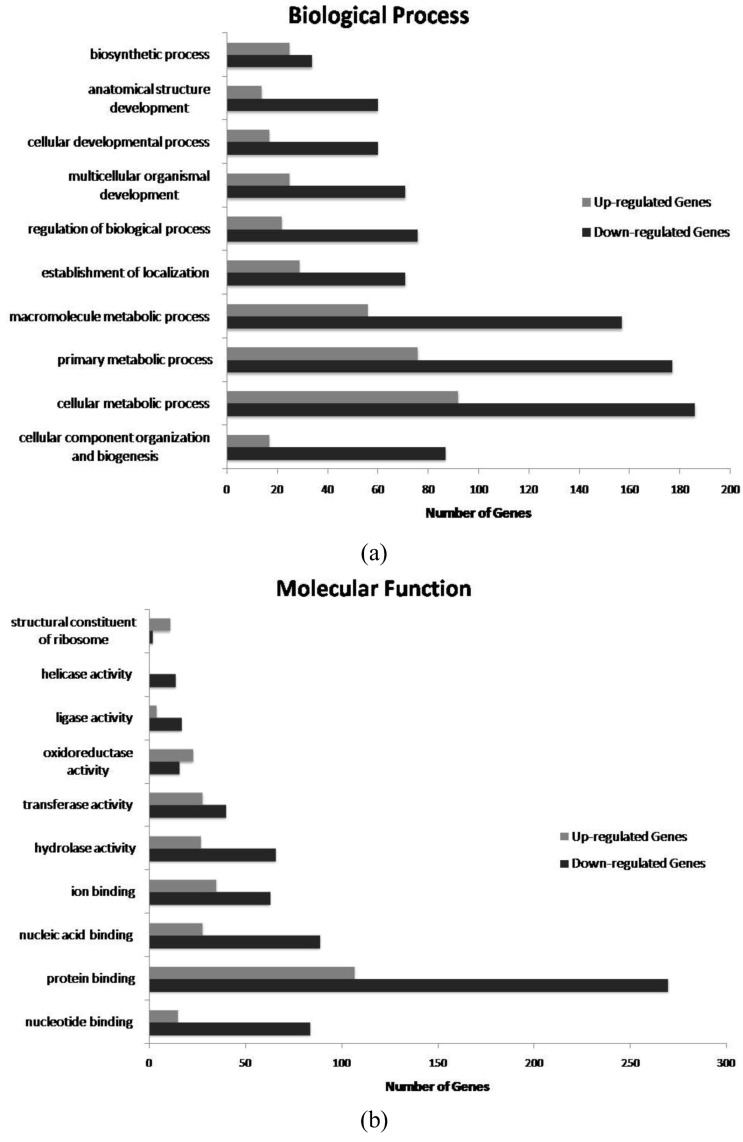
Top 10 Gene Ontology categories representing the major (a) Biological Processes and (b) Molecular Functions affected by dsRNA-GFP treatment during honey bee workers development. For each category (Y axis), the number of down- and up-regulated genes are reported (X axis).

To gain additional functional insights, Gene Ontology (GO) comparisons were performed using “all downregulated genes” *versus* “all upregulated genes” as input. Using GO annotation from *Drosophila melanogaster*, 253 GO terms were recovered for upregulated genes and 508 GO terms for downregulated ones. A number of different metabolic and developmental processes are altered by dsRNA-GFP, and many of the affected genes function as enzymes or binding proteins (Table S4). Although it is difficult to establish trends from the complete dataset (Tables S3 and S4), the most representative GO categories (*top 10*, according to number of genes) are illustrated in Figure 1. As noted above, more genes and processes were downregulated than upregulated by dsRNA-GFP treatment.

Interestingly, the microarray data indicate a perturbation in the metabolism of the major honey bee hormones by dsRNA-GFP treatment. Juvenile Hormone (JH) and 20-hydroxyecdysone (20E) precisely govern virtually all biological processes during the honey bee life cycle, such as morphogenesis, developmental timing, molt and metamorphosis and adult behavior. Levels of juvenile hormone acid methyltransferase (JHAMT, GB10517) transcripts were upregulated in experiment 1, but downregulated in experiment 2, while farnesyl pyrophosphate synthase (FPPS, GB12859) transcripts were upregulated in experiment 2. Both genes are associated with JH synthesis. Juvenile hormone epoxide hydrolase gene (JHEH, GB10771) controls one step of the JH degradation pathway, and its mRNA levels were downregulated in experiment 1. Transcript levels of Ecdysone-induced protein 75 (E75, GB11364) were upregulated in experiment 2. E75 plays roles in molting cycle, cuticle formation, ecdysis and regulation of the ecdysteroid metabolic process. Expression of two storage protein genes, hexamerin 70a (GB30362) and hexamerin 70c (GB13613), were respectively down- and up-regulated in experiment 1. Hexamerin 70a is susceptible to exogenous stimulus, since bacterial infections in adult workers also disrupt its expression [54]. Moreover, honey bee hexamerins function as an amino acids source for metamorphosis, as well as they are potential JH-binding proteins modulating JH action [55]. Accordingly, perturbations in the endocrine system affect both JH- and ecdysteroids-modulated gene cascades and could account partially for the undesired effects observed. 

## 3. Experimental Section

### 3.1. Bees

Three independent experiments were performed. For experiment 1, we used *Apis mellifera carnica* workers maintained according to standard commercial practices at the Chemical Ecology Lab Apiary (Penn State University, State College, PA, USA), while Africanized *A. mellifera* workers from the Apiary of the Department of Genetics (University of Sao Paulo, Ribeirao Preto, SP, Brazil) were used for experiments 2 and 3. In order to obtain age-controlled bees, the queen was caged on a comb and left to lay eggs for 6 h in all experiments. Twenty hours after larval hatching, combs containing second instar larvae were retrieved from the colonies for treatments (see section 3.3. RNAi treatments, sampling and RNA isolation).

### 3.2. dsRNA Synthesis

dsRNA-GFP primers fused with the T7 5’-tail sequence (underlined) were designed (GFP-forward 5’-TAATACGACTCACTATAGGGCGAAGTGGAGAGGGTGAAGGTGA-3’ and GFP-reverse 5’-TAATACGACTCACTATAGGGCGAGGTAAAAGGACAGGGCCATC-3’), and standard PCR was performed using a GFP cDNA clone (File S1) as template. The resulting amplicons were purified with QIAquick PCR Purification kit (Qiagen, Valencia, CA) and used as templates for dsRNA-GFP synthesis using the RiboMax^TM^ Large Scale RNA Production System – T7 (Promega, Madison, WI)protocol. The synthesized dsRNA-GFP products were purified by the TRIzol^®^ LS Reagent method (Invitrogen, Grand Island, NY) and subjected to a denaturation step at 98 °C for 5 min, followed by 30 min at room temperature.

### 3.3. RNAi Treatments, Sampling and RNA Isolation

Because handling and injections may cause stress and affect physiology and survival, we decided to use a non-invasive RNAi protocol described by [23]. In brief, second instar larvae received a single 1 μL dose of a solution containing 5.0 μg (experiment 1) or 0.5 μg (experiments 2 and 3) of dsRNA-GFP, carefully mixed with their natural diet. After treatment, combs were returned to their original colony to develop under natural conditions until the desired stages for analysis: pre-pupae (PP) and light-brown-eyed pupae (Pbl) were sampled for experiment 1, fifth instar spinning phase larvae (L5S2) were sampled for experiment 2 and seven day-old workers (W7d) were sampled for experiment 3. For experiment 3, in particular, newly-emerged workers were maintained in an incubator at 34 °C and a relative humidity of 80% and were provided with water, pollen and sucrose syrup for seven days (W7d). All samples were staged according to criteria developed by [56]. As control groups, age-controlled larvae were left to develop without any treatment (non-treated) and also sampled at L5S2, PP, Pbl and W7d, depending on the experiment. For experiment 1, a total of 16 individuals were collected: four non-treated with PP, four treated with PP, four non-treated with Pbl and four treated with Pbl. For experiment 2, a total of 10 individuals were collected: five non-treated with L5S2 and five treated with L5S2. For experiment 3, a total of 10 individuals were collected: five non-treated with W7d and five treated with W7d. For experiments 1 and 2, each whole-body sample was homogenized in a 1.5 mL tube containing 1 mL of TRIzol (Invitrogen), while experiment 3 used abdomen with adhering fat body and epidermal tissues, which was similarly homogenized as above. All samples were stored at −80°C until the RNA isolation step. Total RNA from each stored sample was isolated following TRIzol manufacturer’s instructions. Subsequently, a purification step was performed using an RNeasy Mini Kit (Qiagen) followed by DNase treatment using an RNase-Free DNase Set (Qiagen).

### 3.4. Microarrays: Hybridization and Data Analysis

Gene expression differences were analyzed using a dye-swap design in which each sample was labeled with both Cy3 or Cy5 probes and hybridized to the honey bee whole genome oligonucleotide arrays supplied by the W. M. Keck Center for Comparative and Functional Genomics at the University of Illinois, Urbana-Champaign. For experiment 1 (which performed a loop design), a total of 200 ng of RNA of each individual (four individuals/sample group) was separately amplified using the Amino Allyl MessageAmp II aRNA Amplification Kit (Ambion, Grand Island, NY), and 5 μg was labeled with Cy3 or Cy5 dye using a Kreatech labeling kit (Kreatech Inc, Durham, NC), for a total of four replicates/sample (20 microarrays). For experiment 2, each group (non-treated or treated) was represented by pools containing 200 ng of total RNA from each individual (totaling 1 μg). One microgram of each pool was separately amplified using the Amino Allyl MessageAmp II aRNA Amplification Kit (Ambion) for which 20 μg was labeled with Amersham Cy3 or Cy5 dye (GE Healthcare Life Science, Piscataway, NJ). Thus, two sets of labeled probes were then hybridized to two arrays. Experiment 3 followed the same procedures described for experiment 2. Slides hybridization, scanning, data normalization and data analysis of experiment 1 followed procedures and steps described in [57,58], while experiments 2 and 3 followed procedures and steps described in [59,60]. The microarray data is available on the Gene Expression Omnibus database (GEO, at NCBI database), according to MIAME standards [61], under the following accession numbers: GSE43193 (experiment 1) and GSE41004 (experiments 2 and 3). 

### 3.5. Bioinformatic Analysis

Sequences from the honey bee Official Gene Set version 1.1 [62] and genome assembly version Amel_4.0 [63] available at BeeBase [64] were used for *in silico* analysis. In addition, some Gene IDs correspond to Expressed Sequence Tags (ESTs) and were recovered from GenBank. Blast searches were performed using NCBI [65] or BeeBase [66] tools. Clustal W [67] was used for multiple alignments of the GFP sequences. Gene Ontology (GO) terms were recovered from *Drosophila* orthologs available at FlyBase [68] and GO classifications and comparisons were performed using Babelomics tool, version 3.2 [69] at level 3. 

## 4. Conclusions

Our results demonstrate that treatment with dsRNA-GFP can have substantial direct and indirect effects on transcript levels of genes associated with a variety of biological processes in developing honey bee workers. Furthermore, it is clear that the molecular effects of dsRNA-GFP exposure can vary depending on the physiological and developmental context; thus, while our studies identified ~1400 affected transcripts, different transcripts may be impacted in different studies. We recommend the use of a second exogenous control in RNAi studies in honey bees in order to better control for the off-target effects of both the control and experimental genes. Further studies will be necessary to determine the non-sequence specific effects of dsRNA-GFP, the effects of dosages and the duration of treatment.

## Supplementary Materials

**File S1**. GFP fasta sequences used in different studies and a multiple alignment of them using Clustal W tool (http://www.genome.jp/tools/clustalw/).

**File S2**. BlastN alignments of dsRNA-GFP sequence with the coding region of some honey bee genes.

**Table S1**. Common honey bee genes perturbed in the three different experiments.

**Table S2**. Opposite gene expression effects triggered by dsRNA-GFP treatment in experiments 1 and 2.

**Table S3.** List of genes up- and downregulated in response to dsRNA-GFP treatment in all 3 experiments. Statistical significance for experiment 1 is provided at FDR>0.05 values, while the adjusted p-value>0.05 is used for experiments 2 and 3. For experiment 1, positive and negative values for fold change mean down- and upregulation by dsRNA-GFP, respectively. For experiments 2 and 3, positive and negative values for fold change mean up- and downregulation by dsRNA-GFP, respectively.

**Table S4**. Molecular functions and biological processes of up- and downregulated genes in response to dsRNA-GFP treatment in all 3 experiments. Classification was based on Gene Ontology terms from *Drosophila* orthologs. Comparisons were performed using Babelomics tool, version 3.2 (http://babelomics3.bioinfo.cipf.es) at level 3.
